# Emigration impact on psychiatric disorders

**DOI:** 10.1192/j.eurpsy.2021.1090

**Published:** 2021-08-13

**Authors:** T. Pengili

**Affiliations:** Psichiatry, University Hospital Center ‘’Mother Theresa’’ Tirana, Tirana, Albania

**Keywords:** Imigration, impact, psychiatric disorders, Imigration, impact, psychiatric disorders, phenomenon

## Abstract

**Introduction:**

Emigration is a widespread phenomenon in our country for the last three decades.Various risk factors for mental disorders are related to emigration,like social-economic status,language,cultural shock,racism etc.

**Objectives:**

The objectives of this study is assess how much of a risk factor is emigration in the development of psychiatric disorders.

**Methods:**

This is retrospective study done on 178 patient charts from The Comunity Mental Health Center Nr.3 in Tirana,of patients who durin the last 20 years had their first episode of mental health disorder durin emigration.

**Results:**

Emigrants before year 2004 had more psychotic disoders,whereas those after that year manifested more mood disorders.The mean age for starting MDD is 35 years old,and the mean age for schizophrenia is 25.Females develope more mood disorders,whereas males manifest more schizophrenia.

**Conclusions:**

Emigration affects deeply mental health,and is a risk factor for developing psychiatric disorders,with females being prone to have mood disorders,whereas males schizophrenia.Schizophrenia start in an earlier age compared to depression.

